# “Effectiveness of minimally invasive injectable modalities in the management of androgenetic alopecia among adults—A systematic review”

**DOI:** 10.1111/jocd.16493

**Published:** 2024-08-23

**Authors:** Vaibhav Kumar, Michael Gold, Anuj Jain, Pranjal Mhatre, Ungku Mohd Shahrin b Mohd Zaman, Rinky Kapoor, Debraj Shome

**Affiliations:** ^1^ Department of Research The Esthetic Clinics Mumbai Maharashtra India; ^2^ Department of Public Health Dentistry Dr GD Pol Foundation YMT Dental College Navi Mumbai Maharashtra India; ^3^ Maastricht University Medical Centre Maastricht University Maastricht Netherlands; ^4^ Gold Skin Care Center, Tennessee Clinical Research Center Nashville Tennessee USA; ^5^ Department of Oral and Maxillofacial Surgery Dr. Hedgewar Smruti Rugna Seva Mandal's Dental College and Hospital Hingoli Maharashtra India; ^6^ Medicine, Bioscience and Nursing Faculty MAHSA University Kuala Lumpur Malaysia; ^7^ USMARI Centre Selangor Malaysia; ^8^ Department of Dermatology, Cosmetic Dermatology and Dermatosurgery The Esthetic Clinics Mumbai Maharashtra India; ^9^ Department of Facial Plastic Surgery and Facial Cosmetic Surgery The Esthetic Clinics Mumbai Maharashtra India

**Keywords:** androgenetic alopecia, biomimetic polypeptides, growth factors, hair regrowth treatments, minimally invasive therapies, QR678

## Abstract

**Background:**

Androgenetic alopecia is the most common cause of hair loss that affects over 50% of the world population. It is a condition that is multifactorial in origin, with no specific causative factor, making treatment an enervating experience for the patient as well as the doctor. In recent times, a number of modalities have been introduced for the treatment of alopecia. However, the evidence supporting them is unstructured and sparse. Therefore, this article aims to explore the current trends in minimally invasive treatments for the management of androgenetic alopecia.

**Methods:**

An in‐depth literature search on injectables used in the treatment of alopecia in PubMed/MEDLINE, Embase, PsycINFO, TRIP Cochrane Library, and Cochrane Skin databases between January 2000 and May 2023 was performed. The studies included were randomized controlled trials, non‐randomized trials, quasi trials, single arm interventions, and cohort studies.

**Results:**

Sixteen of the 1071 studies that were found during the original search were accepted in accordance with the inclusion criteria. Twelve studies assessed the effectiveness of the injectable group by comparing it to a control group consisting of saline, distilled water, and topical minoxidil. In the treatment of alopecia, dutasteride and injectable growth factor formulations achieved clinically significant results.

**Conclusion:**

The usage of injectables and topical medicines to treat hair loss has increased in the recent years. Overall results from clinical investigations, pilot studies, and trials looking at the efficacy and safety of these growth factors in the AGA show satisfactory efficacy.

**Abbreviations**
AGA: androgenetic alopecia
AR: androgen receptors
DHT: dihydrotestosterone
FDA: Food and Drug Administration
FNS: finasteride
ILC: intralesional corticosteroids
RCT: randomized controlled trial
SALT: severity of alopecia tool

## 1 INTRODUCTION

Androgenetic alopecia (AGA), the most prevalent kind of hair loss, commonly known as male or female pattern baldness, affects at least 80% of men and 50% of women, with the frequency rising with advancing years.^1^, ^2^ Even though practicing dermatologists and hair specialists frequently treat it, it might be one of the most difficult conditions to treat because choosing a course of action frequently requires a comprehensive analysis of numerous variables and moral judgment. The most crucial elements to take into account are effectiveness, side effect profiles, practicability encouraging compliance, and treatment cost, especially considering the chronic nature of AGA.

Healthy‐looking hair is correlated with excellent health and beauty in every civilization and culture. Alopecia is characterized as hair loss in the region where hair ordinarily grows. As early as puberty, AGA is shown to be prevalent, affecting 50% of men by age 45–50 and 40% of women by age 65–70.^3^, ^4^ Genetic susceptibility and androgen excess are the causes of AGA. About five million hair follicles are present in each human at birth, and no more follicles develop after birth. The three stages of the hair follicle cycle—anagen, catagen, and telogen—begin in utero. Today, alopecia can be described as the iceberg phenomenon, with the tip of the iceberg, that is seen by the clinicians and the general public, constituting the symptoms of alopecia, and labelling it to be an esthetic concern, and the part of the iceberg underneath the line of horizon being the non visible social, emotional and psychological aspects of the disease and quality of life (QOL) of the person, which remain unsolved.^5^, ^6^

Scalp hair is very important for looks in both women and men. For men, it is an icon of masculinity and for women, it is the crowning beauty of graciously flaunted femininity. The hair follicle has a role in skin tumorigenesis, wound healing, and epidermal homeostasis.^7^ Male pattern alopecia presents as receding frontal hairline, recession in the bi‐temporal region, and thinning on the crown. The severity of hair loss is classified according to Norwood–Hamilton grades I–VII. Female pattern alopecia is widening of the midline region and hair loss on the top with the hairline retained and the severity is classified according to Ludwig's types I–III.^8^

AGA has been treated using a variety of medicinal techniques. However, the approaches and results of medical, surgical, and cosmetic management methods are constrained. AGA impacts a lot of people and their QOL, thus there is a growing need for better therapeutic strategies for its management.^6^ Depression and anxiety are known to be alopecia triggers.^9^ Various stages of the hair cycle can be promoted or suppressed by certain growth agents. Numerous growth factors, including noggin, keratinocyte growth factor, epidermal growth factor (EGF), wingless integration site (Wnt), copper tripeptides, and vascular endothelial growth factor (VEGF), among others, have been proposed for hair growth. The beneficial therapeutic impact of autologous platelet rich plasma (PRP) in AGA is receiving more and more attention. PRP's ability to regenerate follicular epithelium is influenced by the quantity of growth factors produced after activation.^9^, ^10^, ^11^, ^12^ Although many different therapies, such as topical minoxidil, oral antiandrogens, and finasteride, have been tried to treat female pattern hair loss (FPHL), there is still no agreement on the best course of action. Topical minoxidil has a significant negative in that it must be used twice daily for an extended period of time, which can be inconvenient for some patients and irritate their scalps. After stopping the medication, some individuals also experience hair loss.^13^, ^14^ The systemic antiandrogen medication regimen is not advised due to the significant risk of side effects. For the therapy of FPHL, oral finasteride, which has been successful in treating male patients with AGA, is still debatable.

The systematic review focuses on the adult population suffering from androgenic alopecia. The interventions included in the study are the most frequently used minimally invasive techniques and nonsurgical methods for hair restoration and the treatment of alopecia. Most of the studies have a control group that provides a placebo treatment. The outcomes of the studies are measured in standardized tests to assess hair which are phototrichogram, global photographic assessment, video‐microscopic assessment, and patient subjective assessment.

## 2 METHODOLOGY

The current systematic review protocol has been registered on the PROSPERO portal; vide ID CRD42021261016.

This systematic review addresses a focused research question and has been structured in agreement with the Preferred Reporting Items for Systematic Reviews and Meta‐Analyses (PRISMA)‐E 2012 checklist which is an evidence‐based set of items aiming to improve reporting quality of systematic reviews and meta‐analyses.

### 2.1 Search strategy

A primary literature search was conducted using PubMed/MEDLINE, Embase, PsycINFO, TRIP Cochrane Library, and Cochrane Skin databases with search terms (“alopecia”[MeSH Terms] OR “alopecia”[All Fields] OR (“androgenic”[All Fields] AND “alopecia”[All Fields]) OR “androgenic alopecia”[All Fields]) AND (“inject”[All Fields] OR “injectability”[All Fields] OR “injectant”[All Fields] OR “injectants”[All Fields] OR “injectate”[All Fields] OR “injectates”[All Fields] OR “injected”[All Fields] OR “injectable”[All Fields] OR “injectables”[All Fields] OR “injecting”[All Fields] OR “injections”[MeSH Terms] OR “injections”[All Fields] OR “injectable”[All Fields] OR “injectables”[All Fields] OR “injection”[All Fields] OR “injects”[All Fields]).

Given the focus of this article is the use of minimally invasive injectable modalities in patients with alopecia, only studies regarding humans treated with varying standardized and defined grades of alopecia were reviewed for inclusion; clinically relevant systematic reviews, randomized controlled trials, and open studies were considered. Inclusion criteria were studies involving at least one treatment group with the use of synthetic injectables as a treatment modality. Excluded studies included those that were not written in english, involved treatment with hair transplant or a mixed treatment plan, and those that addressed only the mechanism or pharmacodynamics of the injectable treatment. Also, treatment modalities using biological injectables were also excluded. Included studies were graded using the Oxford Center for Evidence‐Based Medicine 2011 Levels of Evidence.

The studies of interest which were included in this systematic review were randomized controlled trials, non‐randomized trials, quasi trials, single arm interventions, and cohort studies. Only those studies utilizing Injectable treatment for AGA on humans have been included in this review. The studies which have been excluded include retrospective studies, narrative reviews, systematic reviews, case reports, technical reports, expert opinions, and descriptive studies. The studies with only their abstracts accessible and those in any language other than english have also been omitted. An attempt was made to distinguish any unpublished studies and to contact the authors of published studies for additional information.

These studies were published between January 2000 and May 2023.

#### 2.1.1 Title and abstract screening

The first phase of the systematic review involved a comprehensive title and abstract screening process to identify relevant studies. A predefined set of inclusion and exclusion criteria was established to guide this process. Two independent reviewers meticulously assessed each study's title and abstract to determine its potential relevance to the research question. Any disagreements between the reviewers were resolved through discussion and, when necessary, consultation with a third reviewer. Studies meeting the initial criteria proceeded to the next phase of full‐text screening.

#### 2.1.2 Full text screening

In the full‐text screening phase, selected studies from the title and abstract screening underwent a thorough examination to assess their eligibility for inclusion in the systematic review. The same inclusion and exclusion criteria established earlier were applied rigorously to evaluate each study's content. Two independent reviewers conducted this process, meticulously analyzing the full text of each study to ascertain its alignment with the research objectives. Discrepancies were resolved through consensus or consultation with a third reviewer. The studies that met the predefined criteria were included in the subsequent data extraction phase.

#### 2.1.3 Data extraction

In the data extraction phase, relevant information from the selected studies was systematically gathered. A structured data extraction form was designed to capture key details, including study characteristics (author, publication year, study design), participant demographics, intervention specifics, outcomes assessed, and study findings. This process was carried out independently by two reviewers, and any disparities were reconciled through discussion or third‐party involvement. Ensuring the accuracy of the extracted data was of paramount importance to maintain the integrity and reliability of the systematic review's findings.

### 2.2 Risk of bias assessment

The quality assessment according to the following quality assessment tools was conducted by two examiners, and it was supervised by a third author for accuracy. The authors resolved any disagreement by consensus. A critical evaluation procedure of each study was done for its internal and external validity and only the articles with good and fair internal and external validity were considered. Critical appraisal was done for each article, depending on the study design, following the Template for Intervention Description and Replication (TIDieR)^15^ checklist and guide, the SPIRIT (Standard Protocol Items: Recommendations for Interventional Trials)^16^ statement, and the CONSORT (CONsolidated Standards of Reporting Trials)^17^ 2010 guidelines.

To assess the risk of bias in pure randomized controlled trial study designs (those classified as such), the Cochrane RevMan 5 software (version 5.4)^18^ was planned to be used for plotting. The standard seven‐point parameters in this tool included checking for random sequence generations, allocation concealment, blinding of participants, blinding of outcome attrition bias, reporting, and other biases. For non‐randomized/ uncontrolled trials, the methodological index for non‐randomized studies (MINORS) scale was used to assess the risk of bias.^19^

## 3 RESULTS

Out of the identified 16 studies, 12 studies are randomized controlled trials which used a control group of saline, distilled water, and topical minoxidil as a comparator to evaluate the efficacy of the injectable group. The studies of interest which were included in this systematic review were randomized controlled trials, non‐randomized trials, quasi trials, single arm interventions, and cohort studies. Only those studies utilizing injectable treatment for AGA on humans have been included in this review.

Summary of study characteristics that were included has been illustrated in Table 1 which includes authors; interventions used, control groups used, age of the participants, parameters assessed, and results and adverse effects.

**TABLE 1:** Table of characteristics.

Study	Intervention	Control	Age group	Assessment	Results	Complications
Abdallah M 2009^20^	Mesotherapy using dutasteride‐containing	0.9% saline	20–50 years	Difference in hair count, professional independent observers assessment Patients' self‐assessment	Increased by 7.739 ± 1.104 hairs. 2.786 and 0.143 in groups I and II, respectively. Mean score = 2.21	Headache and scalp tightness
Doghaim 2018^28^	Carboxytherapy	Injection of distilled water		Clinical assessment, global assessment for hair regrowth. Dermoscopic examination of the lesion. Digital dermoscopic examination of the lesion.	9 (4.5–30) to 5.7 (0–29.7) 50% improvement in the scores 40% improvement 48.5 (9.8–150.2) to 120.8 (16.3–400)	Headache
El Samahy 2021^11^	Lyophilized growth factors (L‐GF)		18 to 40‐year‐old	Trichoscopy, photography score, patient's satisfaction score	89 (65.25–142.25) (80%) showed improvement as following; four patients (20%) showed no improvement Score 1 and score 2 was reported by two patients (10%). Score 3 was reported by nine patients (45%) and score 4 was reported by seven patients (35%).	None
Freund BJ 2010^23^	150 units of botox		19 and 57 years	A change in hair count.	276 (18%)	None
Gajjar 2019^29^	Mesotherapy	Minoxidil	18 and 35	Direct dermoscopy, trichoscan	Variation of hair shaft diameter between the two group was 0.04. We observed a significant increase (*p* = 0.01)	Headache, redness, itching
Kapoor 2020^21^	Intradermal injections of QR678 formulations	PRP	25–50 years	Hair pull test. Video Microscopic assessment.	83% improvement Vellus hair improvement from 52.53 to 40.86 Terminal hair from 64.14 to 78.71 (+14.57)	None
Kapoor R 2018^7^	Intradermal injections of a hair formulation		20–60 years	Hair pull test. Videomicroscopic assessment. Global photographic assessment. Patient self‐assessment. Safety assessments	40% Improvement in the QR group From 167.2 ± 14.4 to 197.1 ± 52.5 Mean—7.5 Improvement in growth of hair (QR678 = 4.4, PRP = 3.2), overall effectiveness of the treatment (QR678 = 4, PRP = 3.8)	None
Kapoor R 2020^4^	QR678®	QR678 Neo®	25–50 years	Videomicroscopic assessment. Global photographic assessment. Patient self‐assessment	Vellus hair improvement from 52.44 to 40.78 Terminal hair from 76.46 to 90.23 (+13.77) 95% improvement 4.5, 4.25, and 4	None
Moftah 2013^38^	Dutasteride‐containing preparation	Saline		Photographic assessment, hair pull test, patient self‐assessment.	Photographic improvement occurred in 62.8% of patients compared with 17.5% in control group (*p* < 0.05), mean number of epilated hairs was significantly decreased (*p* < 0.05) Patient self‐assessment showed statistically significant improvement compared with the controls (*p* < 0.05).	Erythema, pain.
Shome D 2021^22^	Intradermal injection for QR678	Derma roller for QR678	20–70 years	Hair pull test, global photographic assessment, Video‐microscopic assessment, and patient subjective assessment	60% of those in the derma roller group had reduced hair loss. Group A showed significant progress (mean‐7) that was sustained for over a year (mean‐8.8), while Group B mean value was 5.5 at 6 months and improved to 6.7 after a year Vellus hair improvement from 38.54 ± 3.1 to 20.43 ± 2.5 Terminal hair from 65.42 ± 1.7 to 82.50 ± 2.9 4.5, 4.25, and 4	Itchy scalp.
Shon 2020^25^	Botulinum toxin			Phototrichogram image analysis	The mean and standard deviations of hairs per square centimeter at weeks 0, 12, and 24 were 129.61 6 28.05, 129.11 6 28.80, and 136.22 6 33.05, respectively.	None
Singh S 2017^24^	5 U of botulinum toxin		22–42 years	Photography and self‐assessment scoring were done by patients.	Of 10 patients, 8 had good to excellent response on photographic assessment. One patient had poor and one showed fair response to treatment at the end of 24 weeks	No adverse effects
Sobhy N 2013^39^	Mesotherapy	Dutasteride and saline	18–55 years	Trichogram, independent observer assessment of photographs and patients Patients self‐assessment	There is statistically significant increase between anagen hair percent before treatment and after treatment (*Z* = −2.803, *p* = 0.005) Improvement in groups A and B, more in B, as compared to control group. There is no statistically significant difference in patient self‐assessment in the three groups	Mild pain
Uzel BPC 2021^27^	0.5% minoxidil	0.9% saline	18 and 65 years old	Biopsy, trichogram	An increase in the mean T:V ratio in the MG from 2.6 (+ −0.2) to 3.8 (+ −0.5), while in contrast, the PG experienced a decrease in this index from 2.4 (+ −0.2) to 1.7 (+ − 0.2) after treatment. This difference was statistically significant with *p* < 0.001 Increased from 69.9% ± 1.9% before treatment to 78.5% ± 2.0% after treatment. In the PG group, this percentage remained practically stable, varying from 71.8% ± 1.9% before treatment to 72.3% ± 2.2% after treatment	Itching, mild discomfort
Zhou 2020^26^	Botulinum toxin type A		18–60 years	Hair counts, self‐assessment	Hair counts in the BTA + FNS group were 234:01 ± 27:35 root/cm2 and 218:26 ± 30:59 root/cm2 in the BTA group, and the hair counts of both groups were significantly different (*p* < 0.05) Patients in BTA + FNS group reported that they exhibited moderate and marked improvement in fewer symptoms of scalp oil secretion, pruritus, and dandruff	None
Zimber MP 2011^12^	HSC		18–55	Biopsy	HSC‐treated sites demonstrated an increase in hair shaft thickness (6.3% ± 2.5% vs. −0.63% ± 2.1%; *p* = 0.046), thickness density (12.8% ± 4.5% vs. −0.2% ± 2.9%; *p* = 0.028), and terminal hair density (20.6 ± 4.9% vs. 4.4 ± 4.9%; *p* = 0.029).	None

### 3.1 Search results and characteristics of included studies

The PRISMA flowchart for the study search is shown in Figure 1. The search strategies yielded 1071 studies from the databases. Manual searches found 10 additional studies. Of the 1081 studies found through the primary search, 509 and 546 were excluded because of article duplication and title/abstract screening, respectively, and 26 potentially eligible studies were identified. Full texts of all potentially eligible studies were assessed, and 10 were excluded for reasons shown (Table 1). In all, 16 studies met the inclusion criteria (Table 2). The Studies included used injectables as a treatment modality for AGA. All the studies classified the patients for AGA using the Ludwig classification scale or Norwood–Hamilton scale from types I–V. The outcome indicators were measured primarily through a phototrichogram (12 studies) followed by a hair pull test, global photographic assessment, video‐microscopic assessment, and patient subjective assessment and biopsy.

**FIGURE 1:**
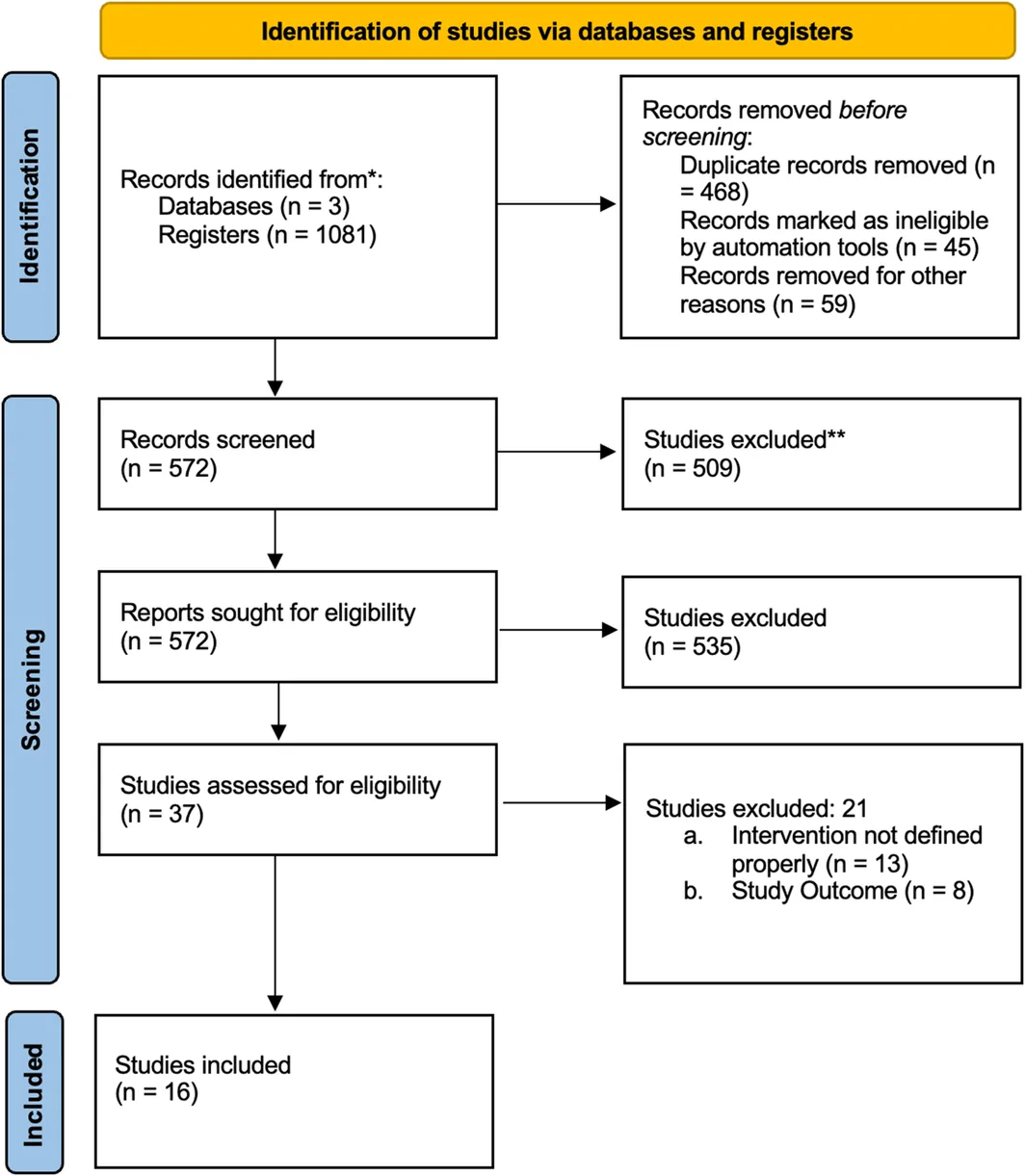
PRISMA flow chart depicting the criteria for identification and screening of the included studies. ** indicate studies excluded by automation tools.

**TABLE 2:** Risk of bias assessment.

Author (year)	Clearly stated aim	Inclusion of consecutive patients	Prospective data collection	Endpoints appropriate to study aim	Unbiased assessment of study end points	Follow‐up period appropriate to study aim	<5% lost to follow‐up	Prospective calculation of sample size	Adequate control group	Contemporary Groups	Baseline equivalence of groups	Adequate statistical analysis	Total minors score
Abdallah M (2009)^20^	2	2	2	2	2	2	2	0	2	2	2	2	22/24
Doghaim (2018)^28^	2	2	2	2	2	2	2	0	2	2	2	2	22/24
El Samahy (2021)^11^	2	2	2	2	2	1	2	0	NA	NA	NA	NA	13/16
Freund BJ (2010)^23^	1	2	2	2	2	2	0	0	NA	NA	NA	NA	11/16
Gajjar P (2019)^29^	2	2	2	2	2	2	0	2	2	2	2	2	22/24
Kapoor R (2020)^21^	2	2	2	2	2	2	0	0	2	2	2	2	20/24
Kapoor R (2018)^7^	2	2	2	2	2	2	0	0	2	2	2	2	20/24
Kapoor R (2020)^4^	2	2	2	2	2	0	0	0	NA	NA	NA	NA	10/16
Moftah (2013)^38^	2	2	2	2	2	0	0	0	1	2	1	2	16/24
Shome D (2021)^22^	2	2	2	2	2	0	0	0	1	2	2	2	17/24
Shon (2020)^25^	2	2	2	2	2	1	0	0	NA	NA	NA	NA	11/16
Singh S (2017)^24^	2	2	2	2	2	1	0	0	NA	NA	NA	NA	11/16
Sobhy N (2013)^39^	2	2	2	2	2	0	0	0	1	1	2	2	16/24
Uzel BPC (2021)^27^	2	2	2	2	2	2	0	0	2	2	2	2	20/24
Zhou (2020)^26^	2	2	2	2	2	0	0	0	NA	NA	NA	NA	10/16
Zimber MP (2011)^12^	2	2	2	2	2	2	0	0	2	2	2	2	20/24

### 3.2 Factor wise comparisons of the treatment modalities

#### 3.2.1 Dutasteride

A study conducted by Abdallah et al. in 2009 to compare the effectiveness and safety of an intradermal injection of a solution containing 0.05% dutasteride to an injection of 0.9% saline using the nappage technique. The dutasteride‐containing treatment outperformed the placebo by a substantial margin (p < 0.05). Three assessment techniques—the difference in hair count, professional independent observers' assessment, and patients' self‐assessment showed that this was the case. The second dutasteride study was conducted by Moftah in 2013 analyzing the effectiveness and safety of mesotherapy for FPHL using a product containing dutasteride. Photographic improvement occurred in 62.8% of patients following mesotherapy with a formulation containing dutasteride compared to 17.5% in the control group. According to the study's findings, mesotherapy with a dutasteride‐containing preparation was a successful, acceptable, and minimally invasive therapeutic option for FPHL, providing improved response for a shorter course of the condition.^20^

### 3.3 Biomimetic Polypeptides injected through mesotherapy

#### 3.3.2 QR678/QR678 Neo

Kapoor and Shome in 2018 tested a novel formulation of biomimetic polypeptides that mimic growth factors in a clinical trial. The goal of this study was to evaluate the effectiveness and safety of the formulation in controlling hair loss and promoting hair growth. A hair formulation was injected intradermally into the scalp skin of 1000 individuals as part of this open‐label, prospective, single‐arm interventional investigation. On the hair pull test, a significant decrease in hair fall was observed in 83% of the patients. The majority of patients exhibited fewer vellus hairs, more terminal hairs, and larger shaft diameters, according to videomicroscopic image analysis. Seventy‐five percent of the patients thought the hair injections were helping them treat their hair loss, and it was even helpful for people who had had hair transplants.^7^ The second study in 2020 by Kapoor compared and assessed the effectiveness of PRP therapy against QR678 therapy in the management of male AGA. After 6 months, the QR678 group experienced a 100% reduction in hair loss, which persisted for a full year. Video microscopic analysis revealed that the QR678 group's hair density, terminal hair density, vellus hair density, and shaft diameter were all noticeably better than the PRP group's (*p <* 0.05).^21^

The third study using the QR678 was done by Kapoor in 2020. The study's objective was to assess the effectiveness of QR678® and QR678 Neo® therapy in the management of female alopecia associated with PCOS. The QR678® formulation is a bio‐engineered recombinant formulation consisting of growth factors, whereas, the QR678 Neo® is a plant‐derived formulation consisting of biomimetic polypeptides that mimic the action of the same growth factors present in the original QR678® formulation. The global photographic evaluation score (mean = 8) showed a significant improvement and was sustained for more than a year. After four sessions, the patients had 8.07 fewer vellus hair, 6.07 more terminal hair, and an average hair shaft diameter that was 0.82 m wider, according to videomicroscopic examination. Following eight sessions, their hair shaft diameter was 2.86 m wider than at baseline, they had 13.77 more terminal hair and 11.66 fewer vellus hair than before.^4^

The QR678 Neo therapy was also evaluated for its effectiveness in the treatment of alopecia areata in a study conducted in 2021. Improvement was assessed by dividing the subjects into two groups. All the subjects were given intralesional corticosteroids in the patches of hair loss. Group A was administered a placebo and Group B was administered with the QR678 Neo® solution. The global photographic assessment showed marked improvement in Group B with a SALT score of 6.6 after 6 months as compared to the SALT score of 38.5 at baseline. Therefore, a combination therapy of intralesional corticosteroids with QR678® Neo solution is a viable treatment option for alopecia areata.

The last study which used the QR678 in 2021 compared the administration of QR678 Neo® hair regeneration therapy for the treatment of AGA in male and female patients using intradermal injection versus derma roller approach. In both groups, a significant reduction in hair fall was seen. In comparison to the derma roller group, all videomicroscopic assessment criteria were better in the intradermal injection group, though not significantly so. Compared to the intradermal group, the derma roller group had more erythema and pain.^22^

#### 3.3.3 Other growth factors

Eslamahy in 2021 assessed the clinical and trichoscopic efficacy and safety of intralesional L‐GF injection in FPHL. A considerable posttreatment increase in all hair parameters was seen by trichologists, along with a significant drop in the number of vellus hairs. All trichoscopic measurements in Ludwig's grade II were significantly different from the baseline after therapy.^11^

### 3.4 Botulinum toxin

Freund in 2010 studied the use of botulinum toxin type A (Botox; Allergan, Inc., Irvine, Calif.) in an open‐label pilot research to treat AGA. The frontalis, temporalis, periauricular, and occipitalis muscles, as well as the periauricular and occipitalis muscles, which surround the head, were given equally divided dosages of 150 units of botox (5 units per 0.1 mL saline) to the subjects at 30 injection sites. The rate of therapeutic response was 75%. Comparable to the outcomes seen with propecia, mean hair counts for the entire group demonstrated a statistically significant (p < 0.001) rise of 18% between baseline and Week 48. In certain subjects, objective hair growth might be seen.^23^ The second study with botulinum toxin was performed by Singh in 2017 in a tertiary care center. Ten male patients with AGA who met the study's inclusion requirements were included overall. A total of 30 locations in the scalp received injections of 5 U of botulinum toxin each. Eight of the ten patients had good to excellent photographic responses. One patient responded to treatment poorly, while another responded to it fairly after 24 weeks. According to their own assessments, 7 out of 10 patients responded well to very well. Two patients responded reasonably and one patient's therapy response was subpar.^24^

In the third study, Shon, in 2020, intradermal BTX injections were given to subjects every 4 weeks for a total of 24 weeks. Each treatment session involved the injection of 30 units of BTX into 20 distinct locations on the balding scalp. The mean 6 standard deviation of hairs per square centimeter at weeks 0, 12, and 24 were 129.61 6 28.05, 129.11 6 28.80, and 136.22 6 33.05, respectively, in an unblinded phototrichogram image analysis. While the number of hair did not substantially increase at week 12 ( p = 14.803), it did at week 24 ( p = 0.012). By week 24, a comparison of the before‐ and after‐treatment photos revealed a noticeable improvement.^25^ The study by Zhou in 2020 examined the effectiveness and safety of botulinum toxin type A (BTA) in AGA patients. Methods. In this trial, 63 individuals with AGA who met the inclusion criteria were treated with BTA injection or BTA injection in conjunction with oral finasteride (FNS). Patients got BTA after every 3 months for a total of four times at 30 places in the scalp where 100 U of BTA was injected into each site. At all‐time intervals, the hair counts in both groups were noticeably higher than they were before to treatment. The BTA + FNS group had higher hair counts than the BTA group after four treatments. The effective rates of BTA and BTA + FNS groups were 73.3% and 84.8%, respectively following four times treatment.^26^

### 3.5 Other treatment modalities

A clinical study by Zimber in 2011 examined the effects of a bioengineered, non‐recombinant formulation made from human cells called hair stimulating complex (HSC), which contained these ingredients, to gauge its ability to stimulate hair growth in men with male pattern baldness. HSC included follistatin, KGF, and VEGF and displayed in vitro Wnt activity. The purpose of the clinical investigation, a randomized, double‐blind, placebo‐controlled experiment, was to determine the safety of the HSC product and its effectiveness in promoting hair growth. During 12 and 52 weeks after treatment, trichoscan imaging analysis of HSC‐treated locations revealed appreciable increases in hair growth compared to the placebo. HSC‐treated sites showed an increase in hair shaft thickness (6.3% ± 2.5% vs. −0.63% ± 2.1%; *p* = 0.046), thickness density (12.8% ± 4.5% vs. −0.2% ± 2.9%; *p* = 0.028), and terminal hair density (20.64.9% vs. 4.44.9%; *p* = 0.029) at the initial 12‐week evaluation period.^12^

Another clinical study conducted by Uzel in 2019 was a randomized, placebo‐controlled trial. The aim of this study was to assess the effectiveness and safety of intradermal injections of 0.5% minoxidil for the treatment of female AGA. In the treated group, there was a substantial improvement in hair loss and volume (*p* = 0.021 and *p* = 0.028, respectively), as well as an increase in the terminal‐to‐vellus hair ratio (*p*0.001) and the proportion of anagen hairs (*p* = 0.048). These findings demonstrate that the safe treatment of female AGA with intradermal injections of minoxidil was superior to placebo (*p*0.001).^27^

Doghaim in 2018 evaluated the clinical efficacy and safety of carboxytherapy in alopecia areata and AGA in two groups; Group I included 40 AA patients (Group IA received carboxytherapy and Group IB control received placebo), and Group II included 40 AGA patients (Group IIA received carboxytherapy and Group IIB control received placebo), and followed up monthly for 3 months. They were evaluated clinically (by assessment of Severity of Alopecia Tool (SALT) score in group I, and Sinclair scale and Norwood–Hamilton scale in group II), by dermoscopy and digital dermoscopy at each visit. Group IA patients showed significant clinical improvement in SALT score and dermoscopic improvement after carboxytherapy and at the end of follow‐up period with significant reduction in dystrophic hair, black dots, yellow dots, and tapered hair coinciding with significant emergence of regrowing hair.^28^

Gajjar in 2019 studied 49 clinically diagnosed AGA males and randomly assigned them to one of two groups—mesotherapy (A) (25) or minoxidil (B)—in a randomized active controlled trial (24). The study found that Group A had significantly more variance in the diameter of the hair shaft between pre‐ and posttreatment than did Group B. Additional dermoscopic, trichoscan, and subjective measuring tools were unable to distinguish between the two groups significantly. These findings imply that mesotherapy for male AGA does not significantly outperform minoxidil.^29^

#### 3.5.1 Risk of bias

The risk of bias assessment table provides a comprehensive evaluation of various clinical studies centered on non‐invasive hair restoration. The assessed parameters include the clarity of the study's aim, inclusion of consecutive patients, prospective data collection, appropriate endpoints aligned with study objectives, unbiased assessment of endpoints, a follow‐up period consistent with study aims, minimal loss to follow‐up, prospective sample size calculation, presence of an adequate control group, use of contemporary groups, baseline equivalence of groups, and appropriate statistical analysis. The evaluation, using a scoring system, yields valuable insights into the methodological strengths and potential limitations of each study. The results reveal notable consistency across many of the evaluated studies in terms of addressing critical aspects like clear study aims, inclusion of consecutive patients, prospective data collection, appropriate endpoints, and unbiased endpoint assessment. Most studies also maintain adequate follow‐up periods with minimal loss to follow‐up, contributing to the robustness of their findings.

However, certain studies exhibit variations in parameters such as the presence of an adequate control group, the use of contemporary groups, baseline group equivalence, and the employment of appropriate statistical analyses. These variations might indicate potential limitations in terms of study design and execution. Nonetheless, it is important to recognize that the overall scores are relatively high across the board, indicating a generally favorable risk of bias assessment.

### 3.6 Assessment of heterogeneity

Heterogeneity of the data was discerned by computing the *I* ^2^ value. For the included studies, this value was found to be 0.88 indicative of a considerable high level of heterogeneity. Thus, owing to the inconsistency, a meta‐analysis could not be carried out.

## 4 DISCUSSION

Dermatologists are adept in treating conditions of the scalp and hair, In today's medical practise, it is important to be aware of hair cosmetics and esthetic techniques as well as the molecular structure and physiological behavior of the hair shaft. Although many hair care treatments are widely available, medical literature is rather scarce, and specialized literature is not readily accessible. In recent years, there has been a paradigm change in favor of evidence‐based treatment and surgery. The paradigm shift has also seen a greater use of injectables and topical medications for the treatment of hair loss.

An evidence‐based strategy involves taking into account the conditions or situations of the patients, identifying knowledge gaps and designing research questions to address those gaps, conducting effective literature searches, and then critically evaluating this research supporting data. Finally, all of this information was gathered to support patient care. Systematic reviews offer a methodical approach to evaluating the available research. The highest degree of evidence for illness prevention, diagnosis, prognosis, and treatment outcomes, according to the Centre for Evidence Based Medicine, is found in systematic reviews.^30^, ^31^ An in‐depth literature search about non‐surgical rhinoplasty revealed numerous studies implementing various study designs. However, there was no systematic review conducted to guarantee the highest possible level of evidence for the minimally invasive techniques mentioned above.

The effects of hair loss on body image, self‐esteem, mental health, and QOL are significant, and they may even trigger psychiatric illness such anxiety and depression. The treatment modalities show a varied range from topical applications to injections of growth factors to the scalp and the hair follicle. AGA's $4 billion in annual global market revenue and 1.8% growth rate illustrate the expansion of the consumer market.^32^

Although minoxidil and finasteride are still the only FDA‐approved and most often used treatments for pattern hair loss, unfavorable side effects have motivated researchers to look for new treatments.

In this systematic review, the authors aimed to identify effective minimally invasive injectable procedures for treating androgenetic alopecia in the adult population. Through a comprehensive literature review, several minimally invasive injectable treatment modalities for alopecia were identified. These modalities include dutasteride, growth factors such as QR678 and intralesional L‐GF, botulinum toxin, HSC formulations containing follistatin, KGF and VEGF, minoxidil, and carboxytherapy. All reviewed studies have consistently shown improvements in hair‐related outcome measures following treatment. For instance, mesotherapy application of dutasteride was found effective in treating alopecia,^33^ showing significant improvement in hair growth compared to control groups. Moreover, growth factors have shown efficacy in the treatment of alopecia. Studies investigating QR678, a formulation containing a combination of growth factors, have demonstrated its effectiveness in improving hair loss conditions. Kapoor and Shome (2018) observed a significant reduction in hair fall among 83% of patients, with the majority experiencing an increase in terminal hairs and shaft diameter, and fewer vellus hairs.^7^ In a separate study on male AGA, QR678 treatment led to a complete cessation of hair loss in all patients after 6 months, with results sustained for a year. Additionally, QR678 treatment resulted in better improvements in hair density, terminal hair density, vellus hair density, and shaft diameter compared to PRP treatment (Kapoor, 2020).^21^ Notably, QR678 has also proven effective in treating female alopecia associated with polycystic ovary syndrome (PCOS).^4^ Botulinum toxin has also been investigated as a treatment for alopecia. Since TGF‐β1 plays a crucial role in AGA, inhibiting its activity could potentially prevent AGA.^34^ Botulinum toxin has been explored for this purpose due to its ability to suppress the secretion of TGF‐β1 from dermal papilla cells (DPCs), similar to its effect on scar tissue fibroblasts.^35^ Studies have reported improvements in hair counts following botulinum toxin treatment by the end of the study period. In addition to the treatments mentioned, interventions such as HSC formulations, intradermal injections of minoxidil, and carboxytherapy have also demonstrated improvements in hair loss patients. Adverse effects observed in the reviewed studies ranged from mild to moderate, including erythema, pain, itching, mild discomfort, headache, scalp tightness, and, in some cases, no adverse effects were reported. Despite these adverse effects, minimally invasive injectable treatments appear to be a viable option for improving alopecia conditions with minimal side effects.

The U.S. Food and Drug Administration (FDA) has approved the use of Minoxidil® and Finasteride® as medications that are currently indicated for AGA. The first medication to be authorized by the FDA for the treatment of AGA in males (1988) and females (1991) was the minoxidil® (pyrimidine derivate) lotion 2%. For males with AGA, Minoxidil® lotion 5% was approved in 1997, and the 5% foam was approved in 2006. By the stimulation of prostaglandin endoperoxide synthase‐1, which raises the level of prostaglandin E2, Minoxidil® lengthens the anagen and increases the HF diameter.^36^, ^37^ Though the effect and results of minoxidil on alopecia are tangible, there is a drawback of it having certain side effects to the patients which include itching, pimples on the face, excessive shedding at the start of the treatment.^27^, ^29^ The Food and Drug Administration (FDA) bodies of various countries are subject to their regional jurisdiction to check the safety of a product before its commercialisation. The QR678 Neo® therapy also has regulatory approval across multiple geographies for its role in the treatment of hair loss.

The 1 mg of finasteride given to male PHL patients for 12 months significantly increased total hair counts for 7.3 percent and 8.99 percent of the patients at 6 and 12 months, respectively (p = 0.001), in a study comparing the effectiveness of standard treatments such as oral finasteride and topical minoxidil with LLLT. In 48 weeks, non‐vellus hair counts significantly increased for 8.84% (*p* = 0.013) and 12.3% (*p =* 0.001) of the 2% and 5% topical minoxidil, respectively.^10^ Though the finasteride targets the genetic causation of alopecia, it does not help in the direct regrowth and nourishment of the active or the damaged follicle, hence most of the treatments suggest it as an adjunct therapy along with the topical agents.^38^

Metwally in 2019 treated patient's individual alopecic patch using intralesional corticosteroids (ILC), intralesional carbon dioxide injection (carboxy therapy), or a combination of both. Assessment was carried out clinically by SALT score, dermoscopically (yellow dots, black dots, tapered hair, regrowing hair), and photographically at baseline, after treatment, and after follow‐up. The SALT score and dermoscopic parameters significantly improved with each treatment course. Analysis of the three treatment modalities showed that the combination regimen resulted in a statistically significant 79.2% hair regrowth, 69.5% improvement after ILC, and 50% improvement after carboxy therapy. The combination regimen also led to the biggest discernible improvement in hair growth after therapy.^39^

According to studies, IGF‐I may play a significant physiological role in regulating hair growth and perhaps the hair growth cycle. Moreover, removing insulin from tissue culture medium may be a practical way to produce a large number of catagen hair follicles for additional in vitro research.^40^ Polypeptides called growth factors control the division and development of numerous cell types. Numerous growth factor families, including the insulin‐like growth factor (IGF), hepatocyte growth factor/scatter factor (HGF/SF), platelet‐derived growth factor (PDGF), transforming growth factor‐beta (TGF‐beta), EGF‐related ligands, and fibroblast growth factors (FGF), have been shown to be essential for the regulation of the hair cycle and hair growth.^41^ The Growth factors included in the systematic review have shown remarkable results with the use of certain growth factors in specific concentrations and dosage to treat AGA.^4^, ^5^, ^21^

### 4.1 Currents trends and innovations in the non‐invasive hair restoration procedures

In the realm of non‐invasive hair restoration procedures, current trends and innovations have ushered in promising advancements, including Regenera and exosome therapies. These methodologies have captured attention due to their potential to stimulate hair regrowth without surgical interventions. Regenera™, for instance, harnesses the power of stem cells to rejuvenate hair follicles, while exosome therapies rely on signaling molecules to trigger regrowth mechanisms. Regenera™ represents a novel approach in the field of non‐invasive hair restoration, centered on the utilization of stem cells to rejuvenate and stimulate hair follicles. Stem cells, owing to their unique capability of self‐renewal and differentiation, offer a potential avenue for addressing hair loss concerns. Regenera's™ mechanism involves the extraction of autologous stem cells, often derived from adipose tissue, and their subsequent enrichment before reintroduction into the scalp. This is theorized to promote cellular regeneration and microenvironmental modulation, thereby encouraging dormant hair follicles to re‐enter the growth phase. While initial observations suggest potential efficacy, the scientific underpinnings and long‐term outcomes of Regenera necessitate further investigation.^42^

In the year 2019, the US Food and Drug Administration issued a Public Safety Notification on exosome products stating the violative nature of “stem cells” products and their unsubstantiated claims in preventing, treating, and curing several diseases and conditions including hair loss. The FDA established that exosomes are regulated as drugs and biological products that are subject to premarket review and approval requirements.^43^

However, while these approaches hold potential, their efficacy remains a subject of debate. Clinical evidence supporting their consistent and substantial hair regrowth claims is often limited. The transient nature of results and varying responses observed among individuals have raised questions about their long‐term impact. Additionally, the costs associated with these procedures can be considerable, making it crucial for individuals seeking hair restoration solutions to approach these innovations with careful consideration and informed expectations. As the industry navigates these innovations, comprehensive research and transparent communication about the limitations and potential outcomes of Regenera™ and exosome therapies are essential for ensuring informed decision‐making. Biomimetic polypeptide/growth factor driven minimally invasive therapies such as the QR678 Neo open an evidence informed vista for predictable treatment outcomes, specifically for androgenetic alopecia and generally for other etiologies of hair loss as well.

## 5 CONCLUSION

AGA is a chronic, crippling illness that adversely affects patients' QOL and leads to significant psychological morbidity. The use of topical FNS and minoxidil for the treatment of AGA has yielded promising results. Intradermal administration of the growth factors/biomimetic polypeptides ‐QR678 in the treatment of AGA is a field of study that is still in its infancy and has only recently been the subject of a few prospective investigations, randomized‐controlled trials, and retrospective analyses of medical records. Carboxytherapy appears to be a viable therapeutic option for patchy AA and may be useful as an adjuvant therapy for AGA, however it will likely take more than six sessions, and adjuvants are advised for maintenance of the outcomes. Overall findings from trials, pilot study and clinical studies examining the effectiveness and safety of these growth factors in AGA demonstrate positive outcomes and non‐inferiority to systemic administration of FDA approved tradional drugs for AGA.

## FUNDING INFORMATION

The authors hereby declare that they have no affiliations with or involvement in any organization or entity with any financial interest or non‐financial interest in the subject matter and materials discussed in this manuscript.

## ETHICS STATEMENT

This study was conducted in accordance to ethical standards, ensuring integrity and transparency. No direct human or animal subjects were involved in this study, and no conflicts of interest are present.

## Data Availability

The data that support the findings of this study are available from the corresponding author upon reasonable request.
